# A case of bullous pemphigoid and renal disease after dipeptidyl peptidase 4 inhibitor administration

**DOI:** 10.1007/s13730-023-00835-1

**Published:** 2023-12-06

**Authors:** Atsuhiko Suenaga, Naoki Sawa, Yuki Oba, Daisuke Ikuma, Akinari Sekine, Masayuki Yamanouchi, Eiko Hasegawa, Hiroki Mizuno, Tatsuya Suwabe, Nobukazu Hayashi, Kei Kono, Keiichi Kinowaki, Kenichi Ohashi, Motoaki Miyazono, Yutaka Yamaguchi, Yoshifumi Ubara

**Affiliations:** 1 Department of Nephrology and Rheumatology, Toranomon Hospital Kajigaya, 1-3-1, Takatsu, Kawasaki, Kanagawa 213-8587 Japan; 2 Department of Nephrology and Rheumatology, Toranomon Hospital, Tokyo, Japan; 3 Department of Dermatology, Toranomon Hospital, Tokyo, Japan; 4 Department of Pathology, Toranomon Hospital, Tokyo, Japan; 5 Department of Human Pathology, Tokyo Medical Dental University, Tokyo, Japan; 6 Okinaka Memorial Institute for Medical Research, Toranomon Hospital, Tokyo, Japan; 7 Department of Nephrology, Saga University Internal Medicine, Saga, Japan; 8 Yamaguchi’s Pathology Laboratory, Chiba, Japan

**Keywords:** Diabetic nephropathy, Dipeptidyl peptidase-4 inhibitor, Glomerular endothelial cell injury, Bullous pemphigoid

## Abstract

A 62-year-old man with type 2 diabetes was admitted because of a decrease in estimated glomerular filtration rate from 72 to 17.5 mL/min/1.73 m^2^ in 10 years and development of widespread bullous skin lesions. His hemoglobin A1c level had been maintained at 6.0–7.0% for 10 years with a dipeptidyl peptidase (DPP)-4 inhibitor. Skin biopsy showed typical bullous pemphigoid, and kidney biopsy showed tubulointerstitial nephritis with eosinophilic infiltration and glomerular endothelial cell proliferation. After discontinuing the DPP-4 inhibitor, skin lesions improved, and renal decline slowed. This case indicates that DPP-4 inhibitors can cause not only skin lesions but also renal disease.

## Introduction

Dipeptidyl peptidase-4 (DPP-4) inhibitors are widely used for the treatment of type 2 diabetes (T2D) [1]. Since Skandalis et al. reported on the relationship between DPP-4 inhibitors and bullous pemphigoid (BP) in 2012 [2], many case reports of BP associated with DPP-4 inhibitors have been published in different countries [3]. In addition to their antihyperglycemic effects, DPP-4 inhibitors promote post-injury regeneration of endothelium and myocardium and may inhibit the development of atherosclerosis [4].

We experienced a case of BP during DPP-4 inhibitor use. In addition, the patient also developed nephropathy, which was characterized by progressive renal function decline. After discontinuation of the DPP-4 inhibitor, skin findings improved and the progression of renal failure slowed. We conclude that although DPP-4 inhibitors have an excellent effect in controlling blood glucose in patients with T2D, they may cause not only skin lesions but also renal complications.

## Case report

A 62-year-old Japanese man was admitted to our hospital for evaluation of renal dysfunction and skin lesions. At age 40 years, he was diagnosed with T2D. He was treated by a diabetes specialist with diet and an alpha-glucosidase inhibitor and had very good glycemic management. At age 51 years, the patient started treatment with the DPP-4 inhibitor sitagliptin. At that time, creatinine was 0.87 mg/dL, the estimated glomerular filtration rate (eGFR) was 72 mL/min/1.73 m^2^, and urine protein was 2+ in the qualitative test. In the subsequent 10 years, the hemoglobin A1c (HbA1c) level was maintained at 6.0–7.0%.

At age 61 years, the patient was switched from sitagliptin to vildagliptin, and one month later, widespread bullous eczema appeared. Vildagliptin was discontinued. The patient’s skin lesions were treated with conservative dermatological treatments, such as steroid ointments, and slightly improved. Four months after vildagliptin was discontinued, sitagliptin was started again. One month later, the skin rash worsened, and during the course of treatment, renal dysfunction slowly progressed. Consequently, the patient was admitted to our hospital.

**Fig. 1:**
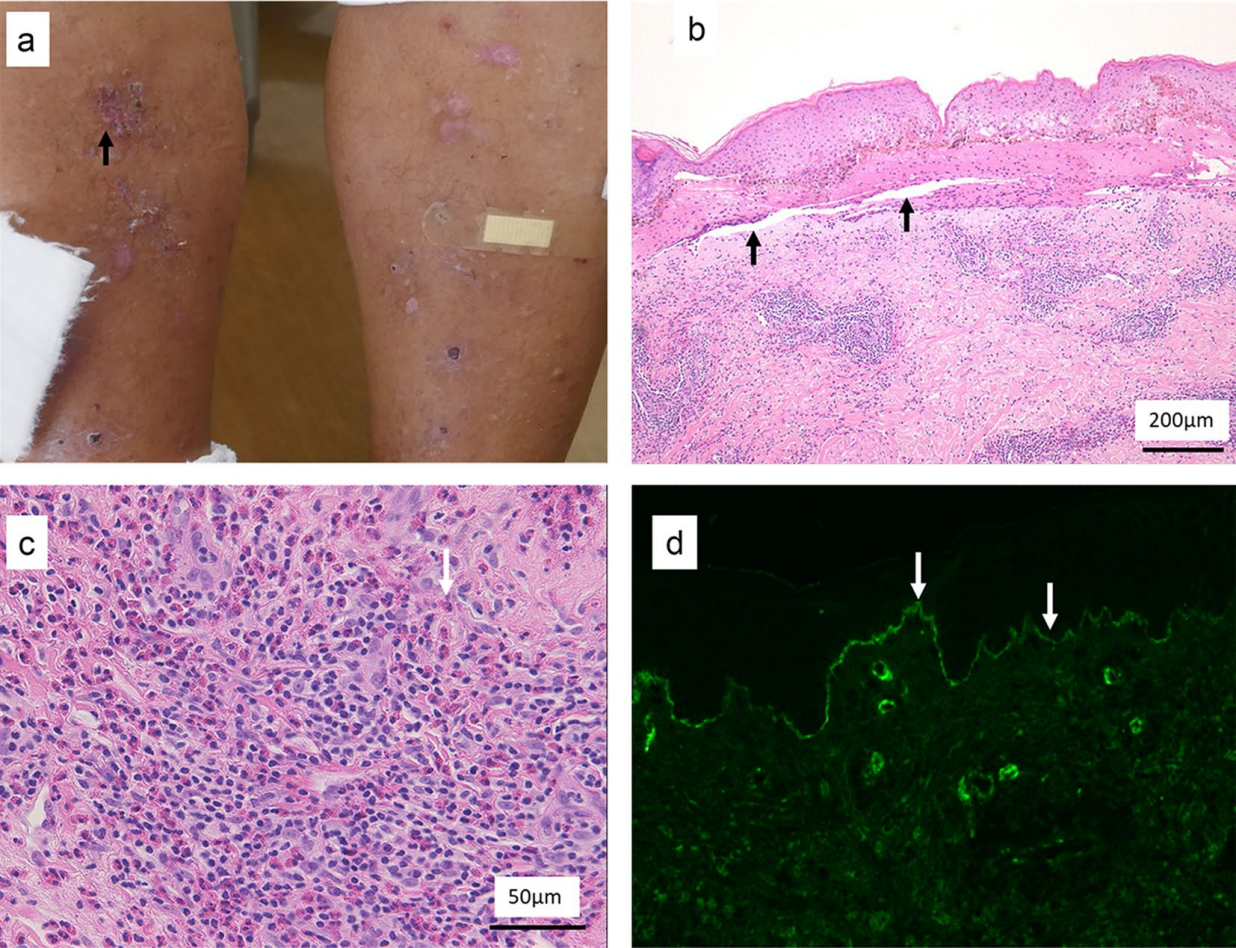
Skin lesions. **a** Numerous bullous lesions on the lower legs. **b** Skin biopsy with subepidermal rip (blisters; arrow); hematoxylin–eosin stain (original magnification × 40). **c** Inflammatory cell infiltrate, mainly eosinophils, in the dermis; hematoxylin–eosin stain (original magnification × 200). **d** Linear deposition of immunoglobulin G (arrow) at the dermo-epidermal junction in immunofluorescence analysis

On admission, the patient was 172 cm tall and weighed 80 kg. His blood pressure was 151/70 mm Hg; pulse rate, 70 beats per minute; and body temperature, 36.1 °C*.* Bullous lesions were present all over his skin (Fig. 1a). The bilateral lower legs were edematous. No abnormalities of the heart, lungs, or abdomen were found.

Laboratory findings were as follows (Table 1): leucocytes, 9100/μL; hemoglobin, 9.9 g/dL; thrombocytes, 211,000/μL; creatinine, 3.05 mg/dL; eGFR, 17.5 mL/min/1.73 m^2^; glucose, 222 mg/dL; HbA1c, 5.9%; triglyceride, 125 mg/dL; total cholesterol, 177 mg/dL; total complement activity (assessed as CH50), 53 U/mL (normal value, > 30 U/mL); complement 3, 74 mg/dL (reference range 86–160 mg/dL); complement 4, 26 mg/dL (reference range 17–45 mg/dL); immunoglobulin G (IgG), 1467 mg/dL (reference range 861–1747 mg/dL); rheumatoid factor, 56 U/mL (reference range < 10.0 U/mL); cyclic citrullinated peptide antibodies, 742 U/mL (reference range: < 4.5 U/mL); and anti-bullous pemphigoid 180 antibody, 5.9 U/mL (reference range: < 9 U/mL). The various other autoantibody tests, including anti-dsDNA antibody, anti-Sm antibody, anti-SS-A/Ro antibody, anti-RNP antibody, anti-desmoglein 1 antibody, and anti-desmoglein 3 antibody, were all negative. Urinary protein excretion was 4.6 g/day, and the sediment contained 1–4 erythrocytes per high-power field. The level of urinary *N*-acetyl-β-d-glucosaminidase was 17.3 IU/day and that of urinary β2-microglobulin, 0.7 mg/day.

**Table 1:** Laboratory fndings on admission

	RR			RR			RR			RR	
*Peripheral blood cell counts*											
WBC	9.1 × 10^3^/μL	3.4–9.2	T-bil	0.7 mg/dL	0.3–1.1	CRP	0.04 mg/dL	870–1700	Anti-DG1-Ab	(–)	
RBC	318 × 10^4^/μL	45–73	AST	32 IU/L	13–33	IgG	1467 mg/dL	5–117	Anti-DG3-Ab	(–)	
Hb	9.9 g/dL	0.0–8.0	ALT	37 IU/L	8–24	IgA	281 mg/dL	110–410	Anti-BP-Ab	5.9 U/mL	< 9.0
Ht	30.9%	13.0–17.0	LDH	261 IU/L	103–190	IgM	120 mg/dL	35–220	*Urine analysis*		
PLT	21.1 × 10^4^/μL	14.1–32.7	ALT	291 IU/L	117–350	C3	74 mg/dL	86–60	Dipstick		
*Blood coagulation*			γGTP	70 IU/L	9–109	C4	26 mg/dL	17–45	Blood	(–)	
PT	87 s	> 75	BS	222 mg/dL	42–127	CH50	53 U/mL	30–50	Protein	(3+)	
APTT	26 s	27–40	HbA1c	5.9%	4.6–6.2	RF	56 U/mL		Sediment		
D-dimer	7.9 μg/mL	< 1.0	TG	125 mg/dL	30–150	Anti-CCP Ab	742 U/mL		RBC	1–4 /HPF	< 4
*Blood chemistry/immunology*			T-CHO	177 mg/dL	< 140	Anti-dsDNA-Ab	(–)		Protein	4.6 g/day	< 0.15
TP	6.5 g/dL	6.9–8.4	Na	138 mmol/L	35–70	Anti-Sm-Ab	(–)		α1MG	20.4 mg/L	1.0–17.8
Alb	3.4 g/dL	3.9–5.2	K	4.0 mmol/L	139–146	Anti-SS-A Ab	(–) Fold		β2MG	0.7 mg/gCr	0.1–1.9
BUN	60 g/dL	8–21	Cl	100 mmol/L	3.7–4.8	Anti-scl70-Ab	(–)		NAG	17.3 IU/gCr	0.8–5.0
Cr	3.05 mg/dL	0.65–1.06	Ca	3.4 mg/dL	101–109	MPO-ANCA	(–) U/mL				
eGFR	17.5 mL/min/1.73 m^2^	> 60	P	3.8 mg/dL	0.0–0.3	PR3-ANCA	(–) U/mL				

Ab, antibody; Alb, albumin; ALP, alkaline phosphatase; ALT, alanine aminotransferase; AMM2A, anti-mimitochondrial M2antibody; AMY, Amylase ANA, antinuclear antibody; APTT, activated partial thromboplastin time; ASMA, anti-smooth muscle antibody; AST, aspartate aminotransferase; BUN, blood urea nitrogen; BS, blood sugar; CCP, cyclic citrullinated peptide; CH50, 50% hemolytic complement unit; Cl, chloride; Cr, creatinine; CRP, C-reactive protein; C3, complement component 3; C4, complement component 4; ds-DNA, double stranded deoxyribonucleic acid; Eo, eosinophil; eGFR, estimated glomerular filtration rate; Hb, hemoglobin; HPF, high power field; Ig, immunoglobulin; K, potassium; LDH, lactate dehydrogenase; LDL-C, low density lipoprotein cholesterol; MPO-ANCA, myeloperoxidase-anti-neutrophil cytoplasmic antibody; Na, sodium; NAG, *N*-acetyl-β-D-glucosaminidase; Neu, neutrophil; PLT, platelet; PT, prothrombin time; PR3-ANCA, proteinase3-anti-neutrophil cytoplasmic antibody; RF, rheumatoid factor; SS, Sjogren syndrome; T-CHO, total cholesterol; TG, triglyceride; TP, total protein; RR, reference range; WBC, white blood cell; α1MG, α1 microglobulin; β2MG, β2-microglobulin; γGTP, γ-glutamyl transpeptidase

Chest X-ray showed an increased cardiothoracic ratio and left-sided pleural effusion. Computed tomography showed bilateral atrophic kidneys, with a long diameter of 84 mm.

## Skin biopsy

Skin samples showed subepidermal blisters (Fig. 1b). An inflammatory cell infiltrate comprising mainly eosinophils was seen in the dermis (Fig. 1c). Immunofluorescence showed the linear deposition of IgG (Fig. 1d) at the dermo-epidermal junction. Bullous pemphigoid (BP) was diagnosed.

## Kidney biopsy

**Fig. 2:**
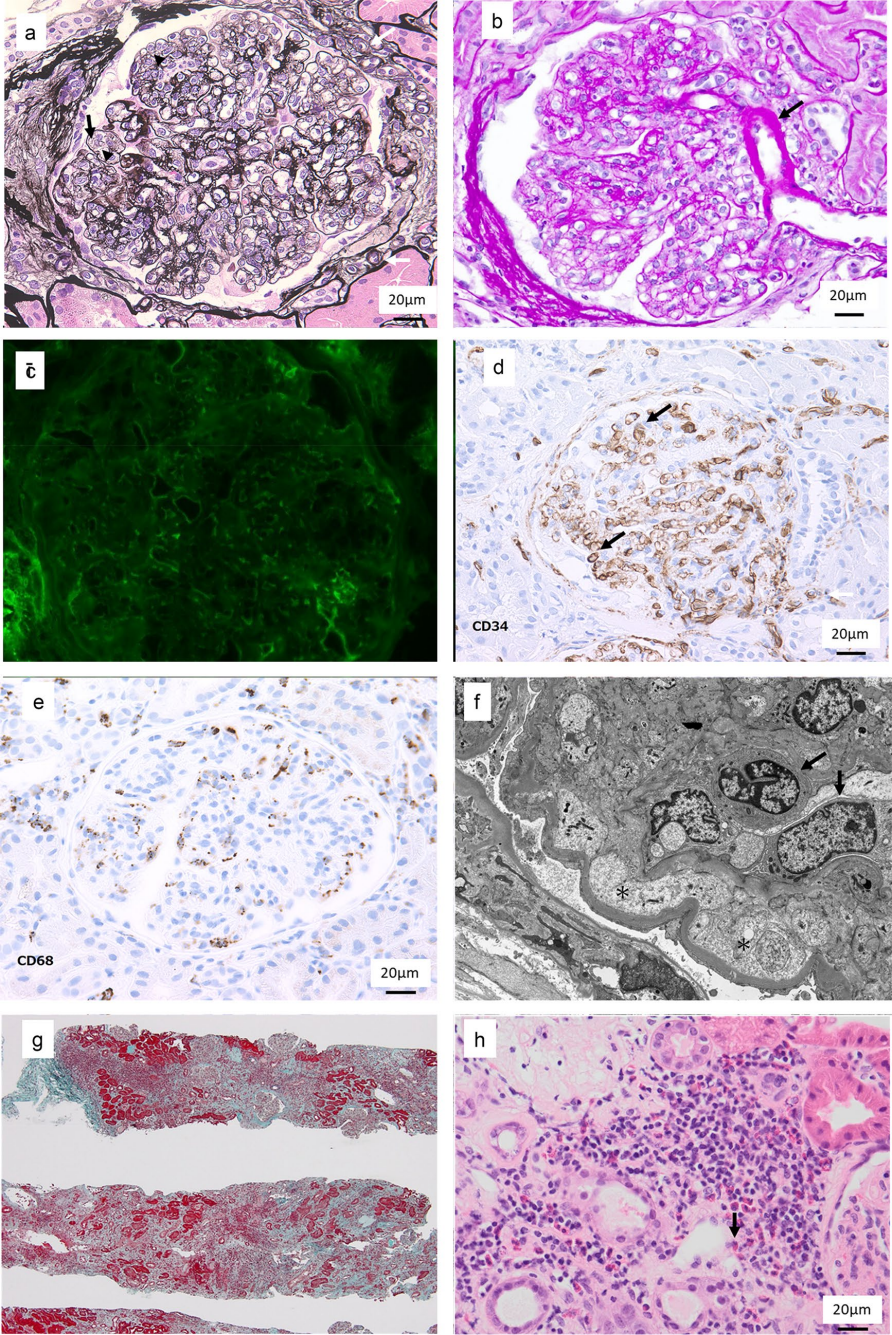
Kidney biopsy. **a** Marked endothelial cell proliferation (arrow head), glomerular basement membrane duplication (black arrow), and lobular structure, with polar vasculosis (white arrow); periodic acid methenamine silver stain (original magnification × 400). **b** Mild arteriolar hyalinosis (arrow); periodic acid methenamine silver stain (original magnification × 400). **c** No staining for immunoglobulin G in immunofluorescence microscopy. **d** CD34-positive endothelial cell proliferation (arrow) in immunohistological analysis. **e** Scattered CD68-positive cells in the glomerulus. **f** Preserved glomeruli with endothelial proliferation (arrow) and subendothelial space enlargement (*) in electron microscopy. **g** Interstitial fibrosis and inflammatory cell infiltration occupying approximately 70% of the cortical area; Masson trichrome stain (original magnification × 40). **h** Eosinophil infiltration (arrow); hematoxylin–eosin stain (original magnification × 200)

Light microscopy examination of a biopsy sample containing 46 glomeruli revealed global sclerosis of 12 glomeruli. Marked endothelial cell proliferation, glomerular basement membrane duplication, and lobular structure were characteristic findings in the preserved glomeruli (Fig. 2a). Nodular sclerosis was not seen. Arteriolar hyalinosis was mild (Fig. 2b), but interlobular arteries showed moderate fibroelastosis. Polar vasculosis was observed (Fig. 2a). Interstitial fibrosis and tubular atrophy with focal moderate infiltration of inflammatory cells, including lymphocytes, plasma cells, and massive eosinophils (Fig. 2h), occupied approximately 70% of the cortical area (Fig. 2g). Immunofluorescence microscopy showed no staining for IgG (Fig. 2c), IgA, IgM, C3, C4, or C1q. Electron microscopy of preserved glomeruli showed endothelial proliferation, subendothelial space enlargement, and partial foot process effacement, but electron-dense deposits were not detected. The glomerular basement membrane was thickened (600–800 nm) (Fig. 2f). Immunohistological analysis also showed CD34-positive endothelial cell proliferation (Fig. 2d). These findings reconfirmed the diagnosis of class 2A diabetic nephropathy according to Tervaert’s pathologic classification [5]. However, the biopsy sample was characterized also by tubulointerstitial nephritis with eosinophilic infiltration and by glomerular microangiopathy with endothelial cell proliferation, findings that are not typical for diabetic nephropathy.

## Clinical course

**Fig. 3:**
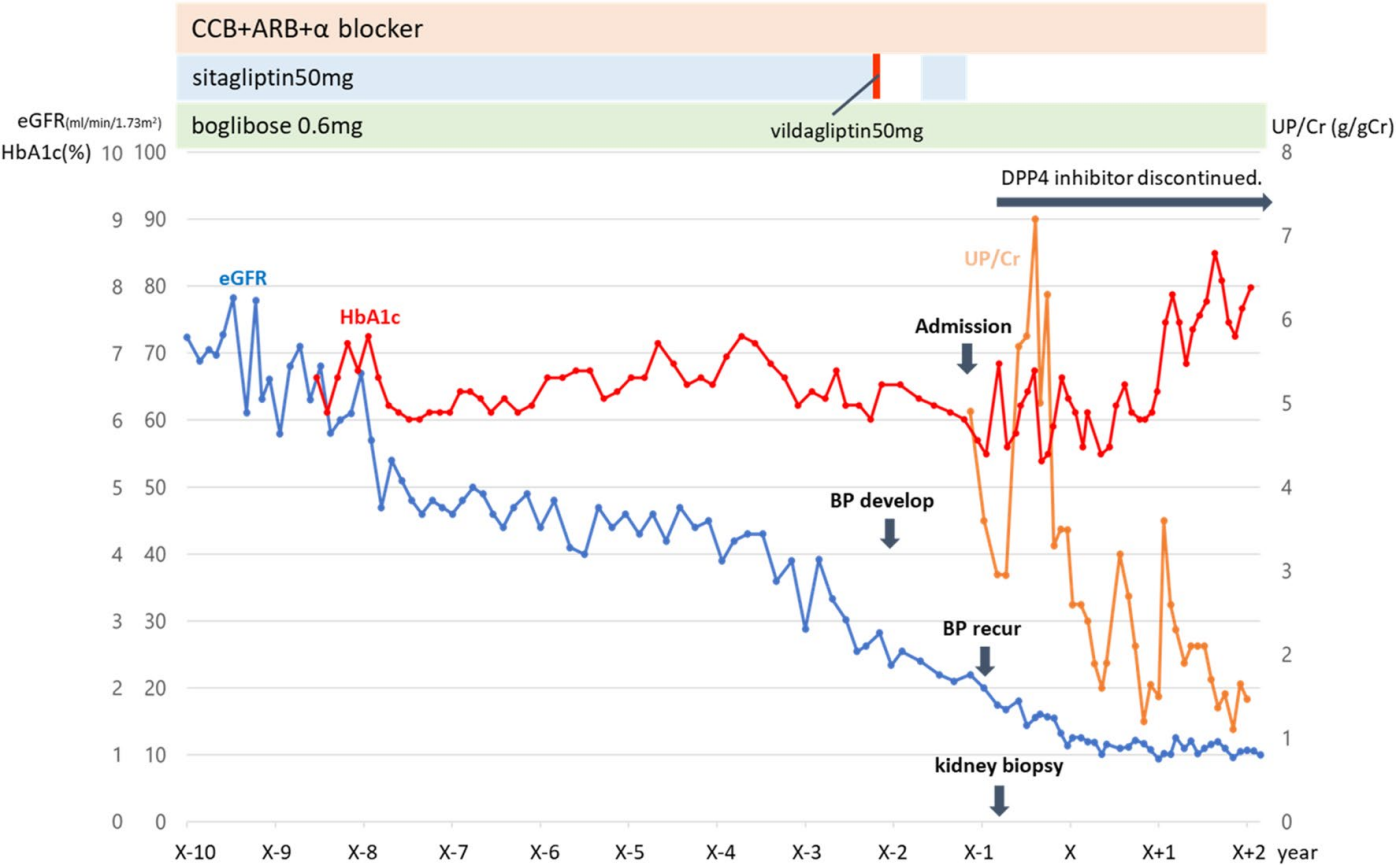
Clinical course. *ARB* angiotensin II receptor blockers, *BP* bullous pemphigoid, *CCB* calcium channel blockers

Sitagliptin was immediately discontinued because it was considered to be a potential cause of the skin manifestations and nephropathy. Post-hospitalization nutritional dietary treatment with restriction of salt intake to 6 g/day and water intake to 1 L/day and continuous use of angiotensin II receptor antagonists and calcium channel blockers led to a body weight loss of 4 kg and a reduction in urinary protein to approximately 1.5 g/day, indicating slowing of the progression of renal failure (Fig. 3). The skin lesions also subsided with continued conservative dermatological treatment with steroid ointments. Immunosuppressive drugs, including steroids, were not administered, as the discontinuation of sitagliptin and the conservative management described above had reduced not only the skin lesions but also the exacerbation of renal function.

## Discussion

Although the patient had good glycemic control for the 10 years after starting treatment with a DPP-4 inhibitor, renal dysfunction progressed over time, and the appearance of skin lesions led us to speculate that the skin and renal lesions might be related to DPP-4 inhibitor treatment. Characteristic BP lesions have been reported as skin lesions caused by DPP-4 inhibitors [2, 3]. However, to our knowledge, renal lesions associated with DPP-4 inhibitors have not been reported to date. Of relevance is that the characteristics of DPP-4 inhibitor-induced renal injury seen in this patient differ from those seen in typical T2D nephropathy. Therefore, when reviewing this case, the reports by Mise et al. on T2D nephropathy are useful [6, 7]. According to these reports, the mean creatinine level of patients with T2D diagnosed with class 2A diabetic nephropathy is 1.2 mg/dL, mean eGFR is 56.9 mL/min/1.73 m^2^, and mean urinary protein is 1.99 g/day. A comparison of these values with those in the present patient shows that the patient’s renal function was clearly impaired and his urinary protein level was higher than that of patients with diabetic nephropathy class 2A.

The present case was also characterized by excessive tubular damage, mainly tubulointerstitial nephritis with eosinophilic infiltration. These characteristics were presumably related to a drug-induced allergic reaction. The glomerular lesions with endothelial cell proliferation were different from typical T2D nephropathy. Brenner et al. reported that DPP-4 inhibitors may promote regeneration of endothelium and myocardium after injury, thereby inhibiting the onset of atherosclerosis, i.e., they suggested that the proliferation of endothelial cells may protect against atherosclerosis [4]. Many other reports also support this idea [8]. This concept appears to apply to the present case because the hyalinization of the arterioles was mild considering that the patient had a long history of T2D and DPP-4 inhibitor was presumably protective against the development of arteriolar lesions. On the other hand, DPP-4 inhibitor may induce endothelial cell proliferation in the glomerulus, which may lead to pathological conditions other than atherosclerosis.

The DPP-4 inhibitor-induced glomerular lesions we reported are similar to the glomerular lesions reported by Eremina et al. as VEGF inhibitor-induced thrombotic microangiopathy [9]. A similar drug-induced mechanism seems to be involved.

Bullous pemphigoid developed after switching from sitagliptin to vildagliptin in this patient. This is in accordance with Arai et al.’s report. Who reported that vildagliptin is actually more likely to cause bullous pemphigoid among several DPP4 derivatives. However, the fact that bullous pemphigoid, which once improved after vildagliptin was discontinued, reappeared on resumption of sitagliptin may be a common feature of DPP4 derivatives [10].

The following discussion is useful because DPP-4 inhibitors have been associated not only with bullous pemphigoid but also with specific nephropathy. The functional role of DPP-4 inhibitors in organ homeostasis or pathology can be divided into two: enzyme-dependent or enzyme-independent mechanisms. In an enzyme-dependent role, the accumulation of CXCL12, a substrate of DPP-4, may be involved not only in the pathogenesis of pemphigoid but also in renal disease. The enzyme-independent role of DPP-4 inhibitors may be implicated in the endothelial proliferation observed in kidney biopsies. It has been suggested that DPP-4 inhibitors have potential effects on endothelial integrin and VEGF signaling pathways. DPP-4 inhibitor has shown to block DPP-4-integrin interaction and then induce VEGF receptors alterations. Glomerular endothelial proliferation was seen when VEGF signaling was too much stimulated. This altered level of integrins and VEGF signaling are also seen in bullous pemphigoid [11].

In conclusion, we experienced a patient with BP and renal impairment after DPP-4 inhibitor administration. After discontinuation of the drug, the skin lesions resolved and the renal decline slowed. The cause of the renal impairment was tubulointerstitial nephritis with eosinophilic infiltration and glomerular microangiopathy with endothelial cell proliferation.

## Data Availability

Data sharing is not applicable to this article as no datasets were generated or analyzed during the current study.

## Abbreviations

BP: Bullous pemphigoid
DN: Diabetic nephropathy
DPP-4: Dipeptidyl peptidase-4
T2D: Type 2 diabetes

## References

[1] Remm F, Franz W, Brenner C. Gliptins and their target dipeptidyl peptidase 4: implications for the treatment of vascular disease. Eur Heart J Cardiovasc Pharmacother. 2015;2:185–93. 27533760 10.1093/ehjcvp/pvv044

[2] Skandalis K, Spirova M, Gaitanis G, Tsartsarakis A, Bassukas ID. Drug-induced bullous pemphigoid in diabetes mellitus patients receiving dipeptidyl peptidase-IV inhibitors plus metformin. J Eur Acad Dermatol Venereol. 2012;26:249–53. 21466592 10.1111/j.1468-3083.2011.04062.x

[3] Tasanen K, Varpuluoma O, Nishie W. Dipeptidyl peptidase-4 inhibitor-associated bullous pemphigoid. Front Immunol. 2019;10:1238. 31275298 10.3389/fimmu.2019.01238 PMC6593303

[4] Brenner C, Franz WM, Kühlenthal S, Kuschnerus K, Remm F, Gross L. DPP-4 inhibition ameliorates atherosclerosis by priming monocytes into M2 macrophages. Int J Cardiol. 2015;199:163–9. 26197403 10.1016/j.ijcard.2015.07.044

[5] Tervaert TW, Mooyaart AL, Amann K, Cohen AH, Cook HT, Drachenberg CB, Ferrario F, Fogo AB, Haas M, de Heer E, Joh K, Noël LH, Radhakrishnan J, Seshan SV, Bajema IM, Bruijn JA, Renal Pathology Society. Pathologic classification of diabetic nephropathy. J Am Soc Nephrol. 2010;21(4):556–63. 20167701 10.1681/ASN.2010010010

[6] Mise K, Hoshino J, Ubara Y, Sumida K, Hiramatsu R, Hasegawa E, Yamanouchi M, Hayami N, Suwabe T, Sawa N, Fujii T, Ohashi K, Hara S, Takaichi K. Renal prognosis a long time after renal biopsy on patients with diabetic nephropathy. Nephrol Dial Transpl. 2014;29(1):109–18. 10.1093/ndt/gft349 PMC3888309 24151019

[7] Mise K, Yamaguchi Y, Hoshino J, Ueno T, Sekine A, Sumida K, Yamanouchi M, Hayami N, Suwabe T, Hiramatsu R, Hasegawa E, Sawa N, Fujii T, Hara S, Sugiyama H, Makino H, Wada J, Ohashi K, Takaichi K, Ubara Y. Paratubular basement membrane insudative lesions predict renal prognosis in patients with type 2 diabetes and biopsy-proven diabetic nephropathy. PLoS ONE. 2017;12(8):e0183190. 28813476 10.1371/journal.pone.0183190 PMC5557586

[8] Yang CJ, Fan ZX, Yang J, Yang J. DPP-4 inhibitors: a potential promising therapeutic target in prevention of atherosclerosis. Int J Cardiol. 2016;202:797–8. 26476032 10.1016/j.ijcard.2015.08.056

[9] Eremina V, Jefferson JA, Kowalewska J, Hochster H, Haas M, Weisstuch J, Richardson C, Kopp JB, Kabir MG, Backx PH, Gerber HP, Ferrara N, Barisoni L, Alpers CE, Quaggin SE. VEGF inhibition and renal thrombotic microangiopathy. N Engl J Med. 2008;358(11):1129–36. 18337603 10.1056/NEJMoa0707330 PMC3030578

[10] Arai M, Shirakawa J, Konishi H, Sagawa N, Terauchi Y. Bullous pemphigoid and dipeptidyl peptidase 4 inhibitors: a disproportionality analysis based on the Japanese adverse drug event report database. Diabetes Care. 2018;41(9):e130–2. 30002201 10.2337/dc18-0210

[11] Shi S, Srivastava SP, Kanasaki M, He J, Kitada M, Nagai T, Nitta K, Takagi S, Kanasaki K, Koya D. Interactions of DPP-4 and integrin β1 influences endothelial-to-mesenchymal transition. Kidney Int. 2015;88(3):479–89. 25830763 10.1038/ki.2015.103

